# Visualization of photothermal therapy by semiconducting polymer dots mediated photoacoustic detection in NIR II

**DOI:** 10.1186/s12951-023-02243-0

**Published:** 2023-12-07

**Authors:** Xiangwei Lin, Zhourui Xu, Jiangao Li, Hongji Shi, Zhenyu Fu, Yuqing Chen, Wenguang Zhang, Yibin Zhang, Haoming Lin, Gaixia Xu, Xin Chen, Siping Chen, Mian Chen

**Affiliations:** 1 National-Regional Key Technology Engineering Laboratory for Medical Ultrasound, Guangdong Key Laboratory for Biomedical Measurements and Ultrasound Imaging, School of Biomedical Engineering, Shenzhen University Medical School, Shenzhen University, Shenzhen, 518055 China; 2 Center for AIE Research, Shenzhen Key Laboratory of Polymer Science and Technology, College of Material Science and Engineering, Shenzhen University, Shenzhen, 518060 China

**Keywords:** Semiconducting polymer dots, Photoacoustic thermometry, Photothermal, Near-infrared II, Theranostics

## Abstract

Visualization of photothermal therapy mediated by photothermal transduction agents (PTAs) is important to promote individual treatment of patients with low side effects. Photoacoustic detection has emerged as a promising noninvasive method for the visualization of PTAs distribution but still has limitations in temperature measurement, including poor measurement accuracy and low tissue penetration depth. In this study, we developed biocompatible semiconducting polymer dots (SPD) for in situ coupling of photothermal and photoacoustic detection in the near-infrared II window. SPD has dual photostability under pulsed laser and continuous-wave laser irradiation with a photothermal conversion efficiency of 42.77%. Meanwhile, a strong correlation between the photoacoustic signal and the actual temperature of SPD can be observed. The standard deviation of SPD-mediated photoacoustic thermometry can reach 0.13 °C when the penetration depth of gelatin phantom is 9.49 mm. Preliminary experimental results in vivo show that SPD-mediated photoacoustic signal has a high signal-to-noise ratio, as well as good performance in temperature response and tumor enrichment. Such a study not only offers a new nanomaterial for the visualization of photothermal therapy but will also promote the theranostic platform for clinical applications.

**Supplementary Information:**

The online version contains supplementary material available at 10.1186/s12951-023-02243-0.

## Introduction

Photothermal treatment (PTT) is a highly effective and noninvasive therapeutic method that shows enormous potential in cancer treatment [1, 2]. In PTT, temperature is one of the most fundamental indicators. According to the different treatment temperatures, it can be divided into hyperthermia and thermal ablation [3, 4]. The temperature of thermal ablation can reach 50 °C or higher, whereas the temperature of hyperthermia is approximately near 43 °C. Some studies have shown that hyperthermia can sensitize tumors to trigger a specific antitumor immune response [5–7]. Indeed, different thermal doses can lead to different biological effects, such as activating thermally sensitive ion channels, changing enzyme activity, and inducing cell apoptosis or death. Thus, high-precision temperature feedback is required to maximize the photothermal effect on the target lesion and minimize the photothermal damage to the surrounding normal tissues.

Currently there are different methods that can be utilized for temperature monitoring, including thermocouple [8], infrared thermal camera (ITC) [9], fiber-optic temperature sensor [10], ultrasonic thermometry [11], magnetic resonance (MR) thermometry [12] and photoacoustic (PA) thermometry [13–18]. However, thermocouple is an invasive and contact detection method, which also costs response time to reach thermal equilibrium. ITC has fast response to measure the spatial distribution of temperature, but it can only reflect the temperature of the surface rather than the heat source deep in situ. Fiber-optic temperature sensor can be used as an invasive temperature measurement of deep-seated tumors, but still limited by contact and single point measurement. As noncontact measurement methods with high penetration, the accuracy of ultrasonic thermometry and the measurement speed of MR thermometry are still limited, respectively. As is well known, the PA signal is related to the Gruneisen coefficient, which is a function related to the temperature. Therefore, PA thermometry has became an emerging technology, which combines the excellent contrast of optical imaging with the good tissue penetration of ultrasound. The concept of PA thermometry was first developed by Rinat et al. in 2005 [13]. Subsequently, PA thermometry has become a valuable feedback modality in different thermal treatment processes, especially in laser radiation [14–18]. However, PA signals of the tissue itself are very weak due to the low absorption coefficient and are easily affected by noise. Meanwhile, PA signals will change if the optical properties of the tissue change under heating treatment. As a result, weak PA signals not only have low sensitivity to temperature rise but also have poor measurement repeatability at a constant temperature.

With the rapid development of nanotechnology, various nanomaterials with high photothermal conversion efficiency not only can promote PTT with more precise and effective, but also can generate more significant wideband ultrasonic waves. Thus, these nanosized photothermal transduction agents (PTAs) that integrate PTT and PA imaging functionalities have led to new and exciting developments in cancer treatment, which is also known as theranostics [19, 20]. In general, a continuous-wave (CW) laser by a fiber-coupled diode laser system is used for PTT, but a nanosecond-width pulsed laser by a solid-state laser system is used for PAI. Different laser sources will lead to different influences on the photostability of PTAs. For example, noble metal materials such as gold nanorods (AuNRs) are ideally suited for PTT applications. Unfortunately, the PA stability of AuNRs is weak and may suffer from partial melting and reshaping [21, 22]. Compared to noble metal materials, organic molecules such as indocyanine green (ICG) have good stability in PAI, but weak performance in PTT stability [23, 24]. Therefore, the utilization of PA thermometry to accurately quantitatively monitor the temperature rise of PTAs deep in-situ is still a major challenge.

Herein, we fabricated semiconducting polymer dots (SPD) as a new nanosized PTA for a visualizable PTT process, including the distribution of SPD and temperature feedback by simultaneously using the photoacoustic signal of SPD. The SPD exhibits outstanding features, including a high extinction coefficient in the NIR-II window with enhanced penetration ability, high photostability under both CW laser and a nanosecond-width pulsed laser, and low cytotoxicity with tumor enrichment ability. More interestingly, the PA signal of SPD has a high correlation to its temperature with a fast response and good reliability. These features will not only benefit for PTT and PA thermometry in situ coupling, but also promote the theranostic platform for clinical applications.

## Materials and methods

### Chemicals and materials

(2E,2’E)-2,2’-(2,8-bis(2-decyltetradecyl)-1,3,7,9-tetraoxo-1,2,3,7,8,9-hexahydro-dithiolo[4’,5’:5,6]benzo[1,2,3,4-lmn] [1, 3]dithiolo[4,5-f] [3, 8]phenanthroline-5,11-diylidene)bis(2-bromoacetonitrile) (NDTA-2Br) and 2,5-bis(2-butyloctyl)-3,6-bis(5-(trimethylstannyl)thiophen-2-yl)-2,5-dihydropyrrolo[3,4-c]pyrrole-1,4-dione (TDPP-2Sn) were purchased from SunaTech Inc. Poly(ethylene glycol) 2000-distearoylphosphatidylethanolamine (DSPE-PEG2000) was purchased from Xi’an Ruixi Biological Technology Co, Ltd. Pd_2_(dba)_3_, P(o-tol)_3_, and anhydrous toluene were purchased from J&K Scientific Ltd. Methanol, acetone, petroleum ether, and chloroform were purchased from Shanghai Macklin Biochemical Co., Ltd. Nitrogen gas (N_2_) was purchased from Shenzhen Shente Industry Gas Co., Ltd. All reagents and solvents were used without further purification.

### Synthesis of NT polymer

The Pd-catalyzed Stille polymerization was carried out to synthetize a polymer with NIR-II absorption. Briefly, NDTA-2Br (132.3 mg, 1.0 eq.), TDPP-2Sn (96.2 mg, 1.0 eq.), Pd_2_(dba)_3_ (4.67 mg, 0.05 eq.), P(o-tol)_3_ (3.04 mg, 0.1 eq.) and anhydrous toluene (10 mL) were added to a dried Schlenk tube (25 mL). All the above operations were carried out in a glove box filled with N_2_. The reaction medium was stirred for 24 h at 120 °C under N_2_. Afterward, 1.0 mL of concentrated hydrochloric acid was added to quench the reaction and stirred for another 12 h. After cooling to room temperature, the polymer was precipitated into cold methanol (300 mL), filtered, and then dried in a vacuum drying oven. The polymer was successively washed in a Soxhlet extractor with acetone, petroleum ether, and chloroform overnight. The final product was collected from chloroform, precipitated into cold methanol again, and dried under reduced pressure at room temperature to obtain a black solid, namely, the NT polymer.

### Fabrication of SPD

SPD was fabricated by a nanoprecipitation method. Briefly, NT polymer (0.5 mg) and DSPE-PEG2000 (2.5 mg) were first dissolved in dimethyl sulfoxide (DMSO, 0.5 mL) solution followed by sonication for 10 min to obtain a uniform mixture. Then, the mixture solution was injected quickly into 5 mL of ultrapure water under vigorous stirring for 2 min. The as-prepared products were then purified by dialysis (molecular weight cutoff: 100 kDa) against ultrapure water for a day to obtain the SPD solution. Finally, the SPD solution was concentrated by ultrafiltration, and the concentration was quantified according to the standard curve of SPD in DMSO. The collected SPD was dispersed into ultrapure water and stored at 4 °C in the dark.

### Characterization

Nuclear magnetic resonance (NMR) spectra were recorded on a Bruker Avance III 500 MHz NMR spectrometer using tetramethylsilane (TMS; δ = 0 µg/ml) as an internal standard. Gel permeation chromatography (Alliance e2695) was used to evaluate the size range of the polymer. Zetasizer NanoZS90 equipment was employed to record the hydrodynamic size and zeta potential of SPD. Transmission electron microscopy (TEM) images were obtained on a Hitachi HT-7700 electron microscope. Absorption spectra were recorded on an Agilent 4100 spectrometer.

### Measurement of the photothermal effect of SPD

The photothermal response of SPD was investigated on a simple setup in the air. Briefly, a centrifuge tube containing 500 µL of SPD at different concentrations was fixed on an optical holder. Ultrapure water was used as a blank control. 1064 nm CW laser with a 1 W/cm^2^ power density was used to irradiate the sample dispersion from the top. An infrared thermal camera (FLIR A300) was operated for temperature recording. The heat conversion efficiency (HCE) was calculated based on Eqs. 1–4 [9].

1 $$\eta =\frac{{hS({T_{max}} - {T_{ssur}}) - {Q_{dis}}}}{{I(1 - {{10}^{ - {A_{1064}}}})}}$$

2 $${\tau _S}=\frac{{{m_D}{C_D}}}{{hS}}$$

3 $$t= - {\tau _S}\ln \theta$$

4 $$\theta =\frac{{T - {T_{ssur}}}}{{{T_{max}} - {T_{ssur}}}}$$

where $\text{h}$ is the heat transfer coefficient, $\text{S}$ is the surface area of the container, and the value of $\text{hS}$ can be obtained according to the natural cooling curve. ${T}_{max}$ is the steady-state temperature of PTA under laser irradiation while ${T}_{ssur}$ is the surrounding temperature. ${Q}_{dis}$ represents the energy absorbed by the container and solvent. $\text{I}$ is the power of incident laser power, while ${A}_{1064}$ is the absorbance of photothermal agents at 1064 nm. *m*_*D*_ and *C*_*D*_ are the mass and specific heat capacity of the sample dispersion, respectively.

### Theory of PA thermometry

The PA signal of the PTA can be generated based on the thermoelastic effect, if the width of the irradiating pulsed laser meets the thermal and stress confinements [25]. After propagation in the medium, the transducer-detected ultrasonic pressure can be described by

5 $$p=\Gamma {\mu _{\text{a}}}F$$

where Γ is the Grueneisen parameter (dimensionless), *µ*_a_ is the specific absorption coefficient, and *F* is the optical fluence. Here Γ can be expressed as

6 $$\Gamma =\frac{{\beta c_{s}^{2}}}{{{C_p}}}=f(T)$$

where *C*_p_ denotes the specific heat capacity at constant pressure, *β* is the volume expansion coefficient, and *c*_s_ is the acoustic velocity. Because the volume expansion coefficient and the acoustic speed are both dependent on the temperature for water-based and fatty tissues between 10 and 55 °C [15, 26], Γ can be derived as a function of temperature T,

7 $$p=f(T){\mu _{\text{a}}}F$$

Thus, the measured PA amplitude is directly dependent on the temperature if *µ*_a_ and *F* are invariable during heating. Note that here, the temperature refers to the base temperature induced by the photothermal laser, whereas the instantaneous temperature increase due to the thermoelastic effect of the absorber is negligible [27].

### Experimental setup for PA thermometry

The platform had three functional modules, including the PA system, PTT system and sample platform. In the PA system, a Nd:YAG pulsed laser (I-20, Surelite) was used. The pulse repetition rate was 20 Hz with an 8 ns width, and the wavelength was tuned at 1064 nm to ensure both optical penetration and absorption of SPD. The energy of the laser pulse was 18 mJ/cm^2^, which was below the American National Standards Institute (ANSI) safety standard (100 mJ/cm^2^). To ensure the consistency of the laser intensity of each pulse, a photodiode (SM1PD1A, Thorlabs) was used to monitor the energy fluctuation between the pulses. An ultrasound transducer (V309-SU, Olympus) with a fixed 25.4 mm focal depth and 5 MHz center frequency was used to detect the PA signal. The PA signal varying with increasing or decreasing temperature was amplified (DPR300 Pulser/Receiver, Imaginant Inc.) and sampled by a 12-bit data acquisition card (ATS9371, Alazar Technologies Inc.) with a sampling rate of 50 MHz. The custom-complied LabVIEW program and MATLAB were separately used to record and analyze the data. The fixed delay output trigger from the pulsed laser synchronized the laser radiation and the data acquisition. In the PTT system, a 1064 nm CW mode laser (GX-1064, Leishi Inc.) was used, and the energy of the CW laser was 1 W/cm^2^. ITC was used to standardize the temperature rise in the PTT process. In the sample platform, water tank with two optical windows was used. A polyethylene tube with a 0.58 mm internal diameter was attached to the optical window for loading SPD at a 100 µg/ml concentration. The pulsed laser, CW laser and ITC were calibrated to the same area. Different insertions of distilled water, gelatin phantom (with 3% intralipid), and intact skull of a mouse were placed in the PA signal propagation path. The CW laser was switched on/off for multiple short-term or the long-term therapeutic periods for different heating requirements.

For 2D PA signal acquisition in vivo, a multichannel ultrasound data transceiver platform (Vantage128, Verasonics, WA, USA) with a linear array ultrasound transducer (L11-4, Verasonics, WA, USA) was used. The system can realize multiangle plane wave ultrasound imaging in transmit-receive mode with PA imaging functions [28].

### Cytotoxicity evaluation

The in vitro cytotoxicity of SPD was evaluated by coincubation with mouse glioma 261 (GL261), breast cancer (MCF-7), breast cancer epithelial (HS578T), human embryonic kidney 293 (293T) cells, and mouse breast tumor (4T1) cells. All cells were seeded in 96-well plates at a density of 5000 cells per well and grown to nearly 80% confluence. Then, the culture medium was replaced by fresh culture medium containing SPD at various concentrations (0, 0.5, 1, 2, 5, 10, 20, 50, and 100 µg/ml). After 24 h of incubation, the cell viability was determined using a Cell Counting K-8 assay.

### Establishment of the 4T1 tumor xenograft model

BALB/c male mice (NO.44,007,200,082,293) were purchased from the Guangdong Medical Laboratory Animal Center. All animals were kept in the Experimental Animal Center of Shenzhen University Medical School. The 4T1 tumor model was established by subcutaneous injection of 100 µL of 4T1 suspension solution (10^7^ cells/mL) into the right underarm of mice. The tumors grew for approximately 10 days and reached a size of approximately 100 mm^3^ before use. The animal experiments conformed to the guidelines of the University Animal Care and Use Committee, according to protocol No. AEWC-202,300,019.

### In vivo experiments

To study the biodistribution of the SPD, the tumor-bearing mice were intravenously injected with 0.2 mL of SPD at a 100 µg/ml concentration. Ultrasound imaging and 2D PA signal acquisition were performed in tumor locations before or after the injection of SPD. Twenty-four hours after injection, the mice were sacrificed and major organs including the tumor, spleen, liver, intestine, bladder, kidney, lung and heart were harvested. Then the ex vivo imaging of the organs was conducted by ultrasound imaging and 2D PA signal acquisition. To study the performance of PA thermometry in vivo, the tumor-bearing mice were intratumorally injected with 20 µL SPD at a 100 µg/ml concentration. Then, ultrasound imaging and 2D PA signal acquisition were performed in tumor locations with or without CW laser irradiation.

To investigate the biocompatibility, BALB/C mice of six-week-old were injected with SPD of 10 mg/kg or PBS of 200 µL. Each group contains five mice. Then, the experiments were performed, including body weight recording, hemolysis assays, blood routine, blood biochemistry, and histological analysis of major organs (heart, liver, spleen, lung, and kidney), respectively.

### Statistical analysis

Data were presented as means ± SD. Statistical analysis was performed using ANOVA. The statistical significance was examined by Student’s t-test when two groups were compared. Statistical analysis was considered significant differences when P values less than 0.05.

## Results

### Synthesis and characterization of SPD

**Fig. 1:**
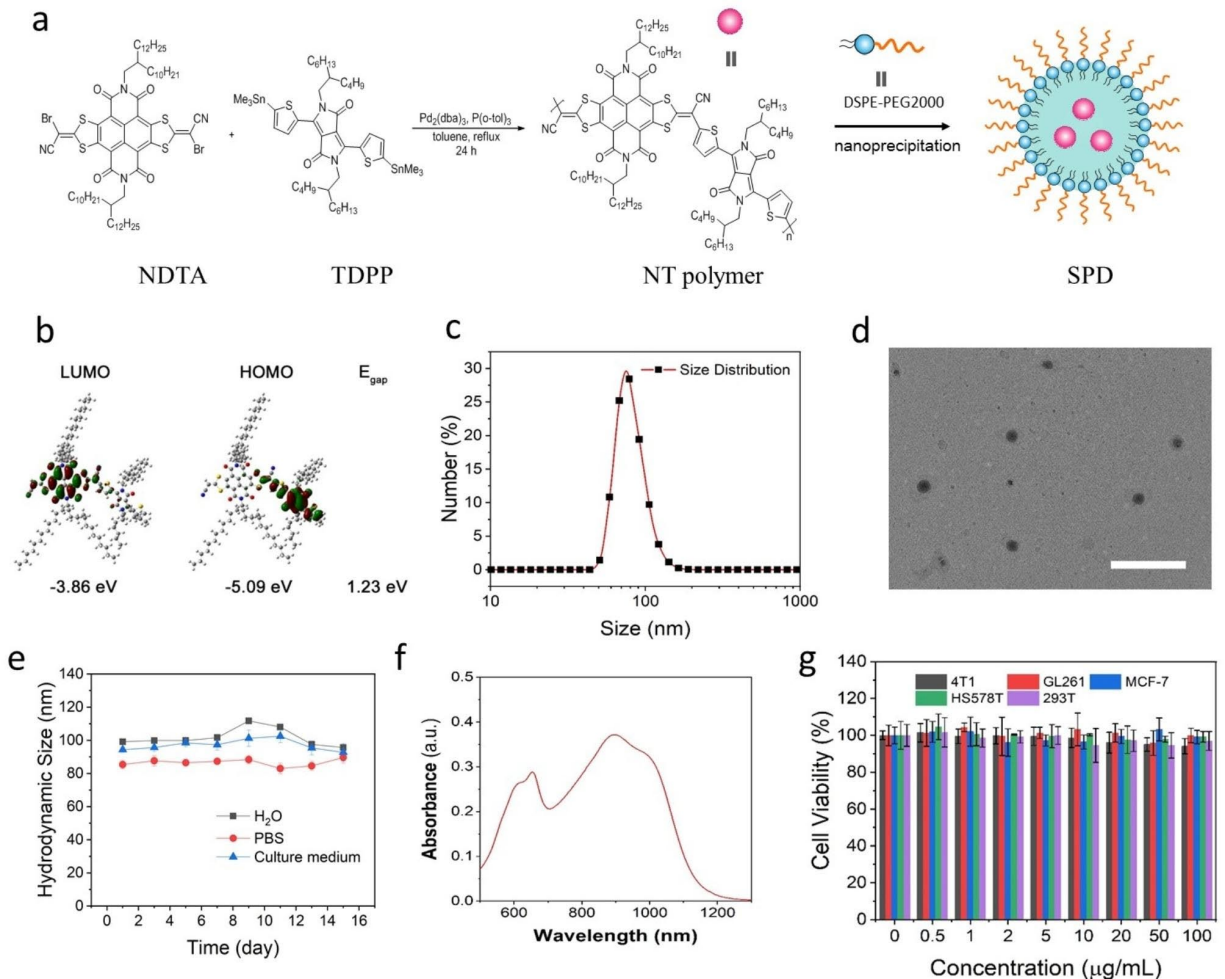
(**a**) Schematic illustration of the synthesis of SPD. (**b**) Calculated molecular HOMO and LUMO orbitals based on density functional theory and a suite of the Gaussian 09 program. (**c**) The hydrodynamic size of SPD characterized by DLS. (**d**) TEM image of SPD. The scale bar is 500 nm. (**e**) The colloidal stability of SPD in PBS serum and culture medium at room temperature for 15 days. (**f**) Absorption spectra of SPD in aqueous solution. (**g**) Cell survival of GL261, MCF-7, HS578T, 293T and 4T1 cells after incubation with different concentrations of SPD for 24 h

Figure 1a shows a schematic illustration of the synthesis of SPD. The structure of SPD is composed of NT polymer as the hydrophobic core and the PEGylated phospholipid DSPE-PEG2000 as the shell. The NT polymer is synthesized by Pd-catalyzed Stille polymerization, with NDTA and TDPP serving as the electron donor and acceptor, respectively. The ^1^H NMR spectrum of the NT polymer is shown in Fig. S1. The number-average molecular weight and polydispersity index of the NT polymer determined by gel permeation chromatography were 13,484 and 2.26, respectively (Fig. S2). Attributed to the strong electron-withdrawing ability of TDPP and the long conjugation length along with the polymer chain, the NT polymer is expected to have strong intramolecular charge transfer, leading to a high extinction coefficient in the NIR-I and NIR-II windows in tetrahydrofuran solution (Fig. S3). Density functional theory calculations were performed to predict and analyze the optical properties of NT using the software Gaussian 09 software and the B3LYP/6-31G(d) method. As shown in Fig. 1b, the electron clouds of the highest occupied molecular orbital (HOMO) are located on TDPP, and the orbitals of the lowest unoccupied molecular orbital (LUMO) are primarily located on the acceptor moiety of NDTA. The band gap was calculated to be 1.23 eV, which is comparable to those of recently reported organic molecules with small band gaps [29]. These calculation results are well in line with the expectations of polymer design.

To endow the hydrophobic NT polymer with water dispersity, thereby applying it to biomedical applications, we capped the hydrophobic core of the NT polymer with DSPE-PEG2000 layer by a well-documented nanoprecipitation method. As shown in Fig. 1c, the hydrodynamic size of SPD is characterized by dynamic light scattering (DLS), which has an average size of approximately 99 nm with a polydispersity index of 0.19. The zeta potential of SPD was about − 29.1 mV (Fig. S4). TEM was further used to study the morphology and size of SPD. As shown in Fig. 1d, spherical SPD has a slightly smaller size of approximately 80 nm, resulting from the dehydration of SPD. In addition, no obvious changes in hydrodynamic size were observed during storage at room temperature for 15 days, indicating that SPD has good colloidal stability (Fig. 1e). Figure 1f shows the absorption spectra of SPD in aqueous solution, exhibiting broad absorption spectra ranging from 500 to 1300 nm. The in vitro cytotoxicity of SPD was evaluated by coincubation with different cells. As shown in Fig. 1g, these cells incubated with different concentrations of SPD for 24 h displayed little toxicity, which is beneficial for further clinical applications.

### PTT and PA performance of SPD

**Fig. 2:**
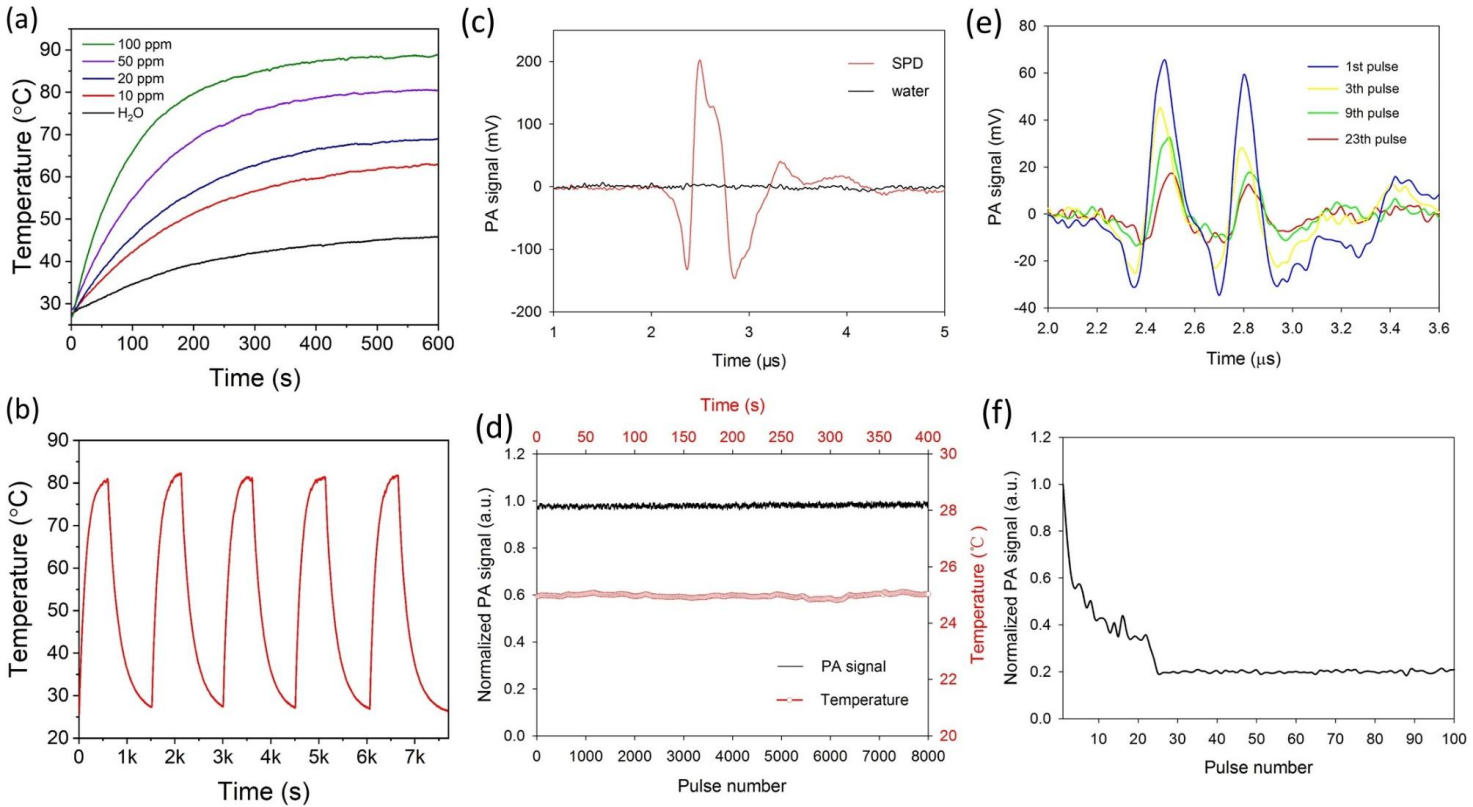
(**a**) Temperature change curves of SPD with different concentrations under the irradiation with a 1064 nm CW laser at a power density of 1 W/cm^2^. (**b**) Thermal stability assay of SPD under the photothermal heating and natural cooling cycles. (**c**) PA signal of SPD and water under a pulsed laser radiation with a width of 8 ns. (**d**) PA stability test of SPD under 8000 laser pulses at the room temperature. The red curve is the corresponding temperature of the SPD solution detected by ITC. (**e**) PA signal of silica-coated AuNRs under different laser pulses. (**f**) PA stability test of silica-coated AuNRs under 100 laser pulses at room temperature

The temperature of SPD was monitored as a function of time under the irradiation with a 1064 nm CW laser by ITC. The laser was switched off when the sample solution reached a steady temperature. As shown in Fig. 2a, the temperature of SPD increased rapidly as the radiation time and concentration increased. In brief, maximum temperatures of 62.7 °C, 69.1 °C, 80.4 °C, and 88.8 °C were realized with SPD concentrations of 10, 20, 50, and 100 µg/ml, respectively. The photothermal conversion efficiency of SPD was determined to be 42.77% (Fig. S5). In addition, the photothermal performance of SPD did not exhibit obvious changes after consecutive heating and cooling cycles, indicating the good photothermal stability of SPD (Fig. 2b). Such good photothermal conversion efficiency of SPD could be explained by the following reasons: (1) the strong electron pull-push effect in NT leads to efficient ICT, resulting in reduced radiative decay and enhanced nonradiative heat generation; (2) the high coplanarity of NT leads to strong molecular π-π stacking, which favors of nonradiative heat generation; and (3) a long π-conjugation length leads to a small band gap, which is beneficial for the ultrafast internal conversion.

The PA performance of SPD was then investigated. As shown in Fig. 2c, under the nanosecond width pulsed laser radiation at room temperature, SPD with a 100 µg/ml concentration produced a 350 mV peak-to-peak PA signal (red line). Figure 2d is the PA signal of SPD under 8000 laser pulses, showing the excellent PA stability of SPD. Meanwhile, the photothermal effect of pulsed laser radiation is negligible, which was detected by ITC (Fig. 2d, black line). Silica-coated AuNRs were used as control, which had a maximum absorption peak at approximately 900 nm (Fig. S6). As shown in Fig. 2e-f, the PA amplitude of silica-coated AuNRs decreased significantly within 23 laser pulses, and the final amplitude was only 20% of the original amplitude.

### The performance of PA thermometry mediated by SPD

**Fig. 3:**
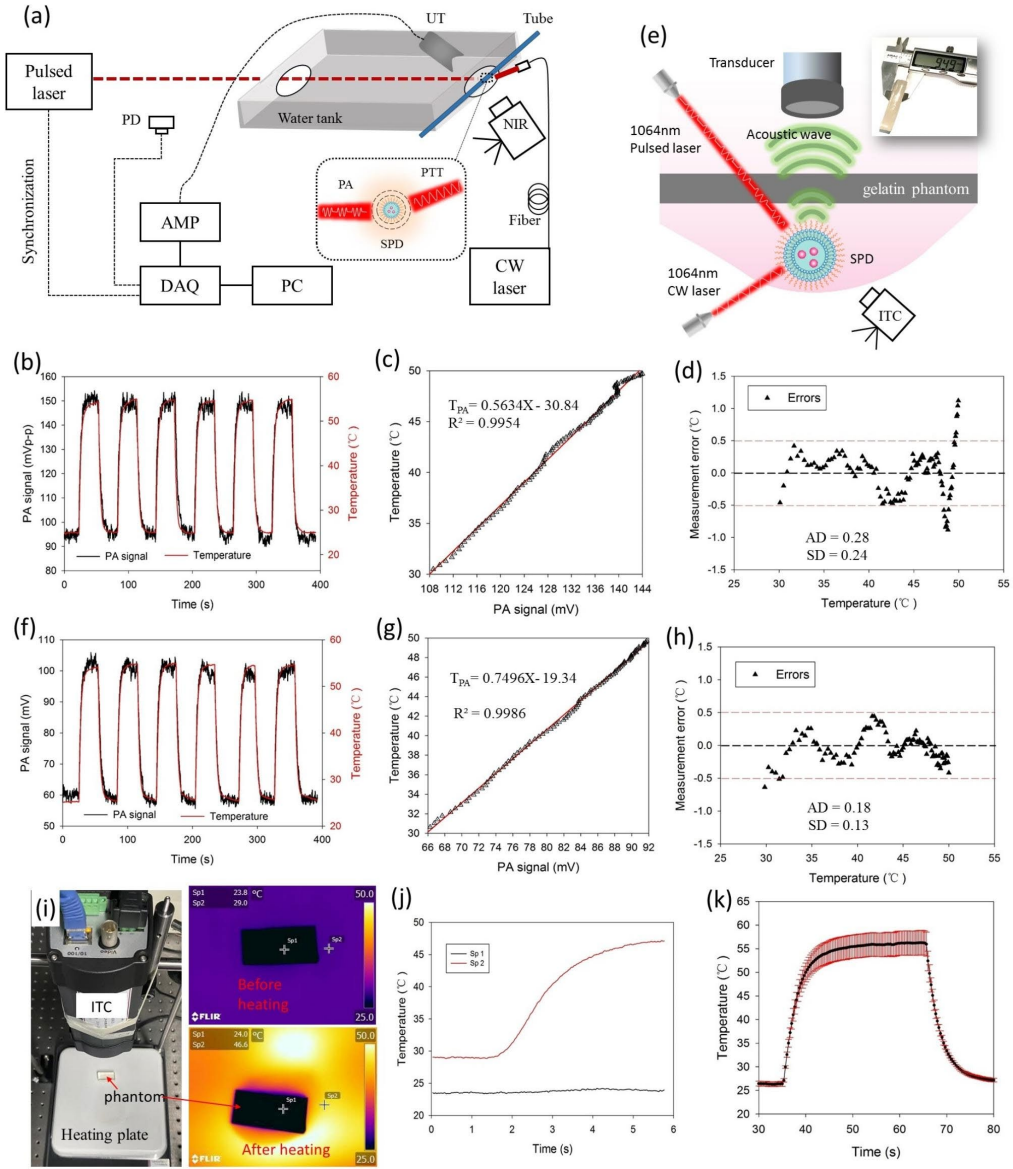
(**a**) Schematic illustration of experimental platform. PD, photodiode; AMP, amplifier; DAQ, data acquisition; PC, personal computer; UT, ultrasound transducer; ITC, infrared thermal camera; CW, continuous-wave; SPD, semiconducting polymer dots. (**b**) The detection results of the PA amplitude and the ITC measured temperature, (**c**) the linear relationship between the PA amplitude and temperature, and (**d**) the measurement errors of the PA thermometry. (**e**) Schematic illustration of the experimental platform with the insertion of a gelatin phantom. (**f**) The detection results of the PA amplitude and the ITC measured temperature with a 9.49 mm gelatin phantom, and (**g**) the linear relationship between the PA amplitude and temperature, and (**h**) the measurement errors of the PA thermometry. (**i**) Control experiment by using ITC to investigate the temperature of a heating plate, which is partially covered with the same thickness phantom. Left is a photograph of the experimental platform. On the right is the thermogram by ITC. (**j**) The temperature changing profile of different monitoring points in the control experiment. (**k**) The average and SD of temperature detected by ITC in a cycle of (**b**)

After proving the photostability of SPD under both CW laser and nanosecond-width pulsed laser, we then investigated the performance of PA thermometry mediated by SPD. As a high-sensitivity and high-resolution temperature detector, ITC can be used as the gold standard. To solve the limitation of ITC for surface inspection only, we constructed a platform for in situ coupling of PTT and PA detection. Figure 3a is the schematic experimental platform. The solution of SPD was equilibrated to room temperature before heating. Then, we measured the amplitudes of the PA signals and temperature by ITC simultaneously. When exposed to the CW laser, the average temperature of the irradiation area of the SPD solution was increased from approximately 25 to 54 °C, as detected by ITC (Fig. 3b). When the CW laser was switched off, the temperature of the SPD solution was quickly cooled down to room temperature for the thermal diffusion with a small sample tube diameter (Video S1, Supporting Information). At the same time, the PA amplitude of the detected area kept synchronously increasing or decreasing in the heating or cooling process, the value of which varied between approximately 91 mV and 151 mV with the insertion of water only. More interestingly, the PA signal followed the actual temperature profile very well in the whole process. In addition, such performance was quite stable in the cyclic testing. As shown in Fig. 3c, a linear relationship between the PA signal and the temperature detected by ITC was observed in the interval of 30 to 50 °C. Thus, the equation between the PA signal and temperature with the insertion of only water can be calculated as: T_PA_=aX + b, where T_PA_ is the temperature transformed from the PA signal, a and b are the weight factors for different insertions, and X is the amplitude of the PA signal. According to the above results, the linear equation was T_PA_=0.5634X-30.84, and a correlation coefficient (R) of 0.9954 was used to assess the goodness-of-fit of the linear model. Figure 3d shows the measurement errors of the PA thermometry, compared with the temperature detected by ITC. The absolute deviation (AD) and the standard deviation (SD) were 0.35 °C and 0.24 °C, respectively.

To verify the deep-tissue penetration in the NIR-II window, we further used a gelatin phantom with a thickness of 9.49 mm as an insertion placed in the PA propagation path (Fig. 3e). As shown in Fig. 3f, under the same CW laser heating conditions, the average temperature of the irradiation area of the SPD solution was almost the same as that before. Meanwhile, a similar trend of the PA signal following the actual temperature profile in the heating or cooling process was observed. However, the detected PA intensity was attenuated after passing through the gelatin phantom, the value of which varied between approximately 59 mV and 101 mV. The linear equation in the interval of 30 to 50 °C was calculated as: T_PA_=0.7496X-19.34, and the R value was 0.9986 (Fig. 3g). The AD and the SD were 0.18 and 0.13, respectively (Fig. 3h). Then, we used ITC to detect the temperature of a heating plate as a control experiment. As shown in Fig. 3i-j and Video S2 (Supporting Information), the ITC has difficulty monitoring the temperature of the heating plate, which is partially covered with the same thickness phantom.

Note that there was a slight fluctuation of the PA signal at the beginning and the end of each cycle. The possible reason is that disordered thermal diffusion is dominant with a relatively small temperature gradient change in the SPD solution. As shown in Fig. S7, the temperature distribution in the irradiation area has differences, which may lead to PA signal fluctuation. Figure 3k shows the average and SD of the temperature of the ROI area detected by ITC in a cycle, which has more significant temperature fluctuations in the plateau phase. We also numerically calculated the heat diffusion and PA signal generation within a two-dimensional heterogeneous medium by using k-waves [30]. Fig. S8 is a simple demonstration of using k-waves for the simulation of heat diffusion. The simulation results show that the SD of temperature in the ROI region increases with increasing temperature, which is consistent with the experimental results.

**Fig. 4:**
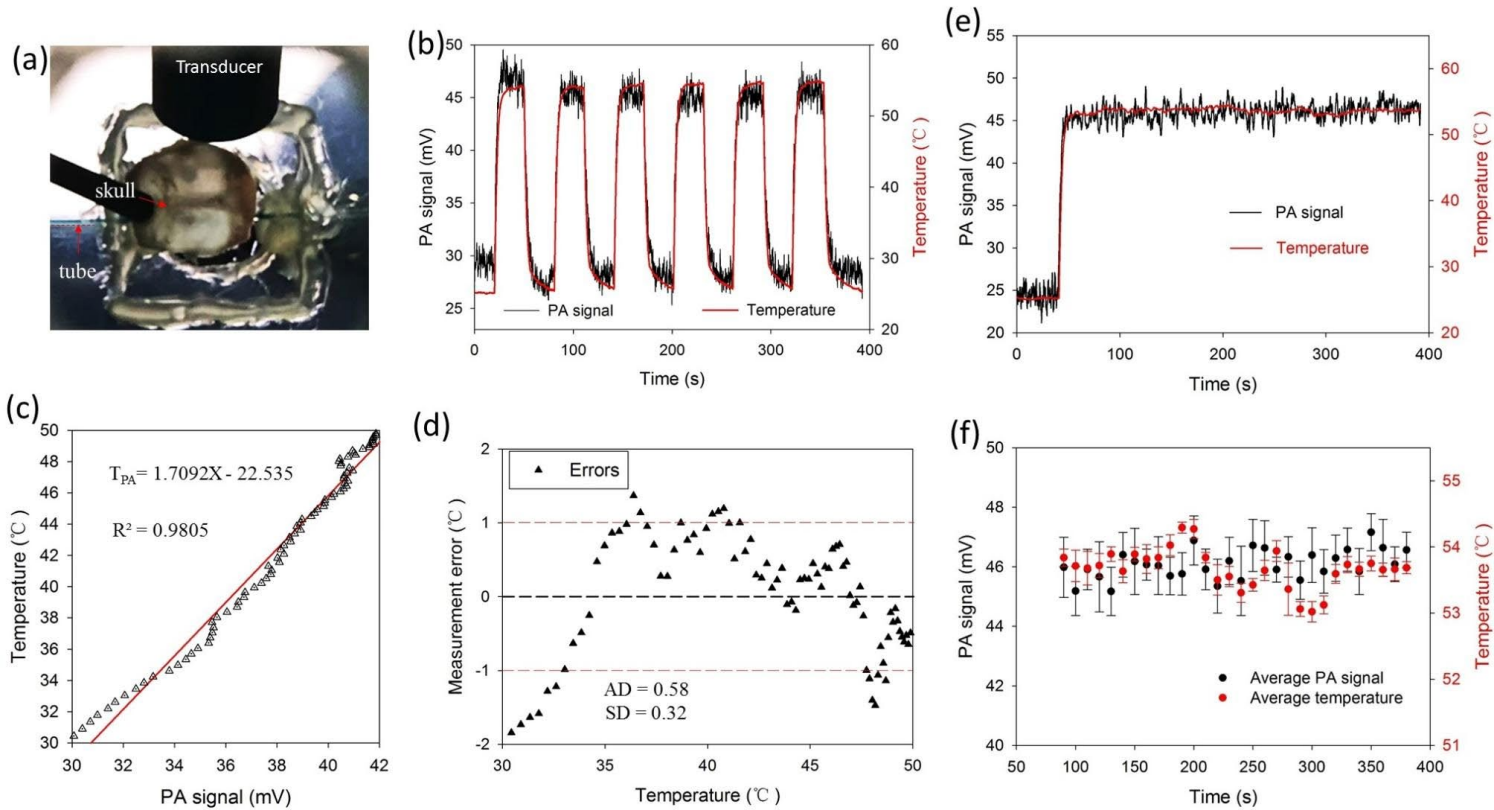
(**a**) Photograph of the partial platform. (**b**) The detection results of the PA amplitude and the ITC measured temperature with skull of mouse, and (**c**) the linear relationship between the PA amplitude and temperature, and (**d**) the measurement errors of the PA thermometry. (**e**) Long-term PA monitoring of the SPD crossing the skull. (**f**) The measurement errors of the photoacoustic thermometry

In addition to the gelatin phantom, the skull of the mouse was used for insertion (Fig. 4a). Because the skull has high optical attenuation and a large acoustic impedance difference, it is more difficult for optical and acoustic transmission. However, as shown in Fig. 4b, a linear correlation between the PA amplitude and the measured temperature can still be observed by using a 1064 nm laser and SPD agents. The detected PA intensity varied between approximately 27 mV and 46 mV. The linear equation in the interval of 30 to 50 °C was calculated as T_PA_=1.7093X-22.535, and the R value was 0.9805 (Fig. 4c). The AD and the SD were 0.58 and 0.32, respectively (Fig. 4d).

Figure 4e shows the results of PA thermometry crossing the skull when the SPD solution was exposed long-term to the CW laser. The results show good responsiveness of PA thermometry mediated by SPD for a long-term PTT process. When the photothermal conversion and thermal diffusion of the SPD solution are balanced, the PA signal and detection temperature by ITC are relatively stable. Thus, we can establish a simple corresponding relationship between the average PA signal and the average actual temperature in the stationary period. Figure 4f is the average value of ten seconds of PA signal and corresponding temperature every 10 s from the 90th second. Based on the data, we obtained an average PA signal in the stationary period of 46.08 ± 0.48 mV, with a corresponding temperature of 53.69 ± 0.29 °C.

**Fig. 5:**
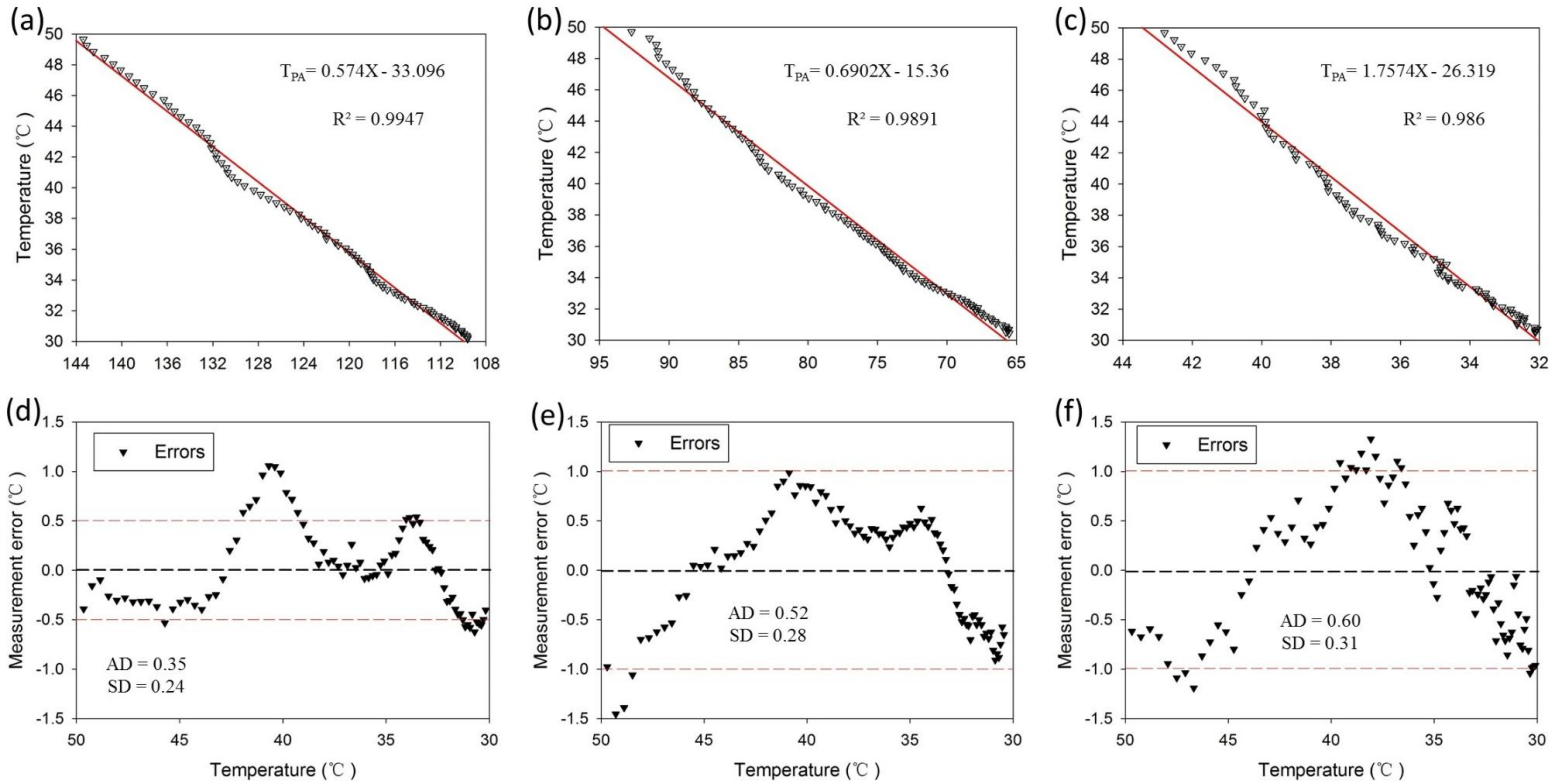
The linear relationship between the PA amplitude and temperature in the cooling process with insertions of (**a**) water, (**b**) gelatin phantom and (**c**) skull. (**d**–**f**) The measurement errors of PA thermometry according to (**a**–**c**)

We further investigated the influence of the temperature change rate on PA thermometry between 30 and 50 °C. In the experiments, it takes 5.20 ± 0.10 s to make the temperature change from 30 to 50 °C in the heating process, compared with that of 4.35 ± 0.09 s from 50 °C to 30 °C in the cooling process. The linear relationships between the PA signal and the temperature detected by ITC from 50 °C to 30 °C with different insertions are shown in Fig. 5a-c. Figure 5d-f shows the corresponding measurement error distributions, and all the related data are listed in Table 1. According to our results, the influence of the temperature change rate on PA thermometry is not obvious. Note that different media will lead to different optical and acoustic attenuations of PA signals while the heat source has the same temperature. Thus, the attenuation coefficient of different media must be considered for the quantitative analysis in PA thermometry. In addition, when establishing the connection equation, it is necessary to consider the case of nonlinearity, which should be established with a piecewise function.

**Table 1:** PA thermometry mediated by SPD.

Insertion	PA temperature equation	Absolute deviation (AD/°C)	Standard deviation (SD/°C)	Correlation coefficient (R^2^)
W/h	T_PA_=0.5634X-30.84	0.28	0.24	0.9954
W/c	T_PA_=0.574X-33.096	0.35	0.24	0.9947
P/h	T_PA_=0.7496X-19.34	0.18	0.13	0.9986
P/c	T_PA_=0.6902X-15.36	0.52	0.28	0.9891
S/h	T_PA_=1.709X-22.535	0.58	0.32	0.9805
S/c	T_PA_=1.757X-26.319	0.60	0.31	0.986

W. P, and S refer to the insertion of water, phantom, and skull, respectively; h, and c refer to the heating process and the cooling process, respectively.

### Visualization of photothermal therapy in vivo

**Fig. 6:**
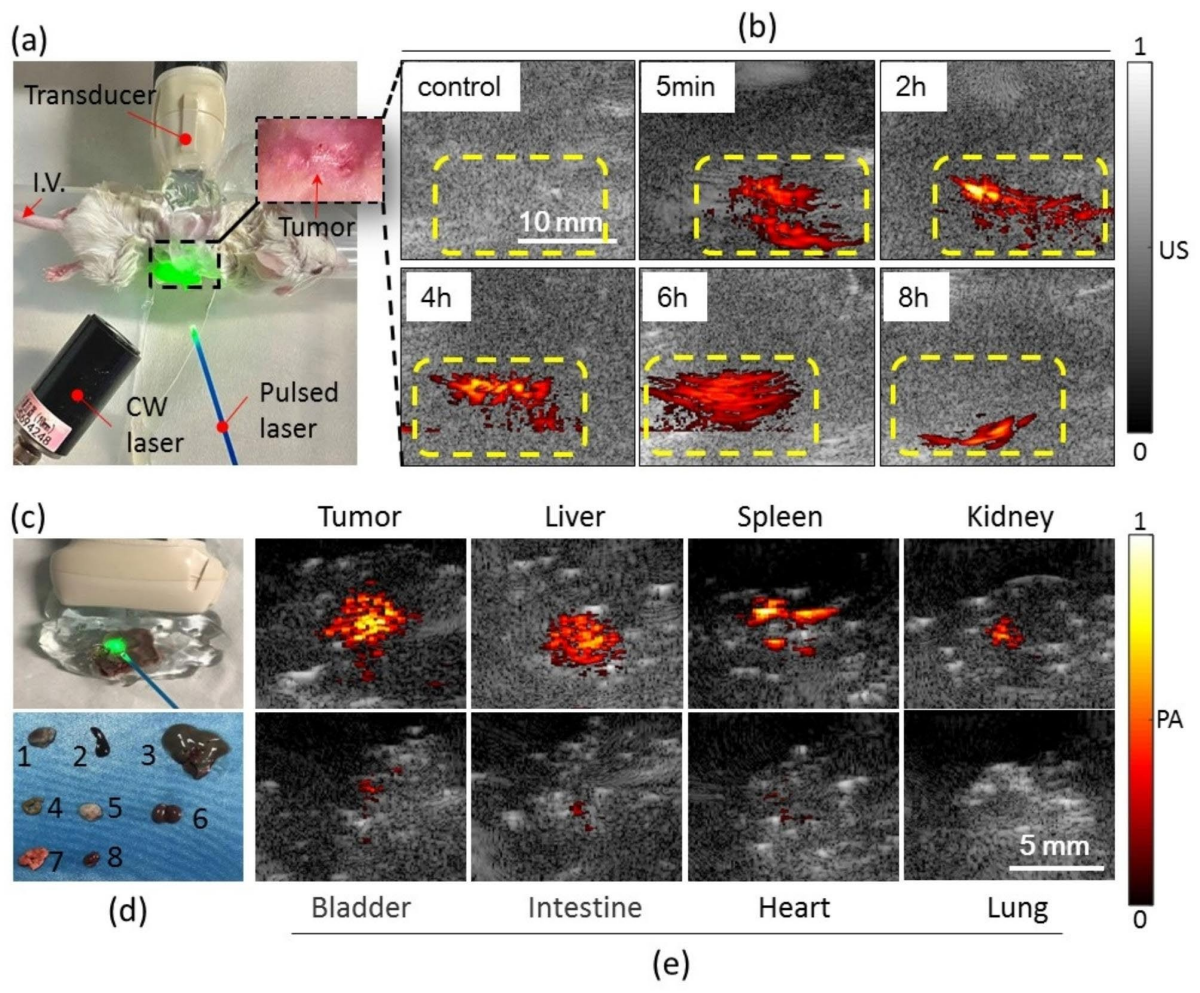
In vivo experiments. (**a**) Photograph of the partial experiment. (**b**) The biodistribution of the SPD in the tumor before or after injection of SPD. The yellow dashed box represents the area containing the tumor. (**c**–**e**) Ex vivo images of the major organs at 24 h after injection of SPD. (**c**) Photograph of the partial experiment. (**d**) Numbers 1 to 8 correspond to tumor, spleen, liver, intestine, bladder, kidney, lung and heart, respectively

To assess the performance of SPD in vivo, a verasonics system with linear array ultrasound transducer was used for 2D PA signal acquisition in the subcutaneous tumor mouse model (Fig. 6a). Indeed, the boundaries of the tumor and organs were not obvious in the ultrasonic grayscale image. However, we can roughly estimate the location of subcutaneous tumor by comparing the spatial structure. As shown in Fig. 6b, before giving an injection, there were few PA signals in the tumor area, indicating negligible absorption of tissues under irradiation with a 1064 nm pulsed laser. At 5 min postinjection through the tail vein, the PA signal of SPD was sharply visualized in the same irradiation area where the signal was enhanced over 40 times. With the extension of time, the PA signal of SPD showed significant enhancement within 2–4 h. After 24 h, ex vivo organ imaging showed that SPD was mainly distributed in the tumor, liver, spleen, and kidneys, with little distribution in the bladder, intestine, and heart (Fig. 6c-e). The results indicated that SPD can aggregate in the tumor region with the enhanced permeation and retention effect.

**Fig. 7:**
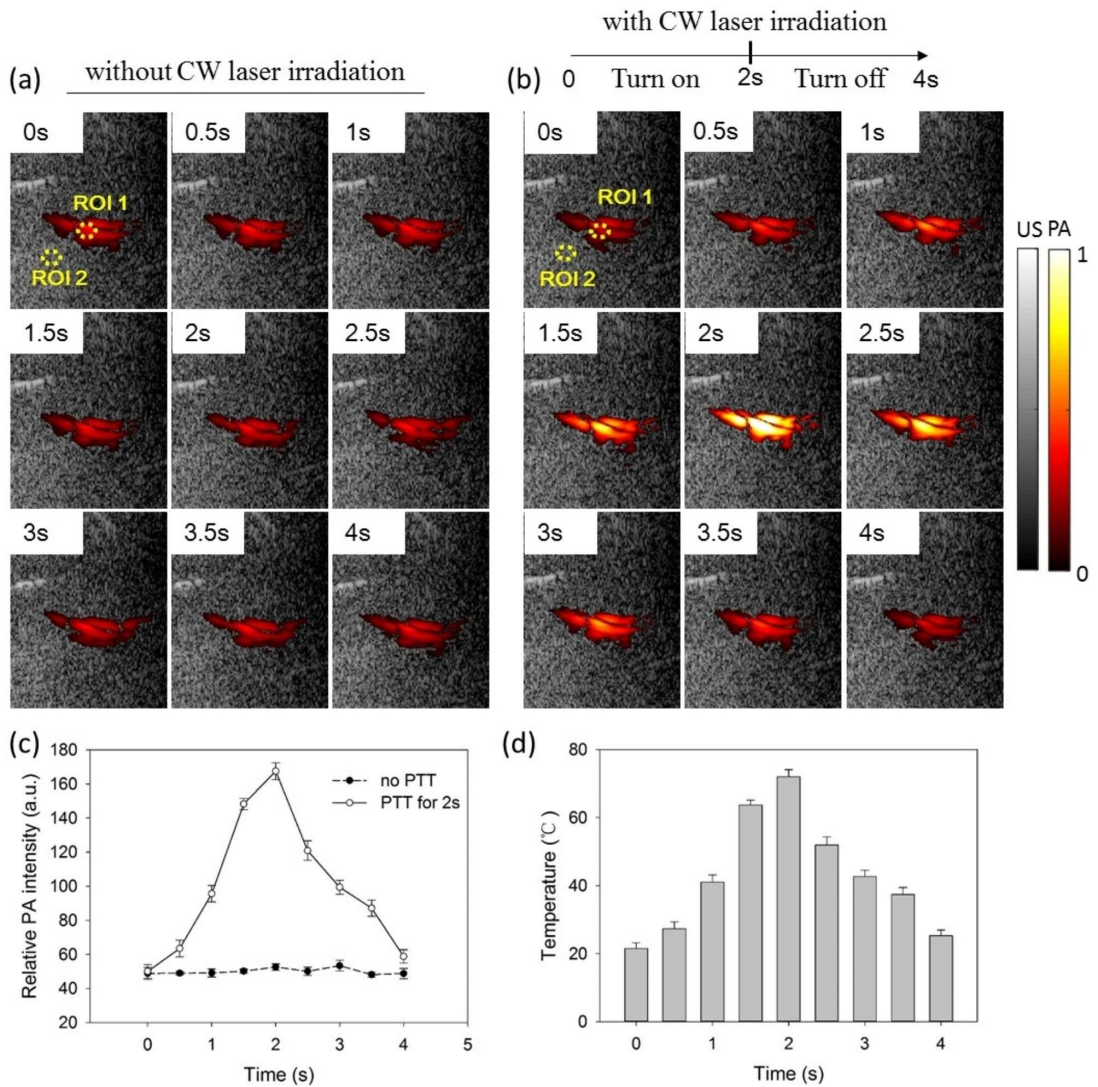
The PA signal of SPD by intratumoral injection before or under irradiation by a 1064 nm CW laser. (**a**) Without CW laser irradiation. (**b**) With CW laser irradiation for 2 s. (**c**) Quantitative analysis of the relative PA signal intensity (the ratio of SPD signal to tissue signal) of intratumoral SPD. (**d**) Quantitative analysis of intratumoral SPD temperature with CW laser irradiation for 2 s

Figure 7a shows the PA signal of SPD by intratumoral injection before irradiation by 1064 nm CW laser. There were no significant changes in signal intensity within 4 s, but there was a little influence on the signal distribution by the respiratory movement of the mouse. However, under a short irradiation cycle by a 1064 nm CW laser, the SPD signal showed a strong correlation following the PTT process (Fig. 7b). As shown in Fig. 7c, the relative PA signal intensity (the ratio of SPD signal to tissue signal) increased from approximately 50 to 167 during the 2-seconds CW laser irradiation, and then was decrease to about 59 during the 2 s cooling process. As the initial temperature of the tumor area can be measured through thermoelectric coupling, the boundary conditions for the PA signal and temperature function of SPD can be obtained. According to Eq. (7) mentioned above, we can simply estimate the temperature of SPD where the changes in the specific absorption coefficient and the optical fluence can be ignored in the PTT process. Figure 7d shows the estimation of SPD temperature in the ROI 1 region from Fig. 7b-c. It is interesting to note that the temperature of SPD was increased from approximately 21.5 to 72.0 °C during the 2 s CW laser irradiation. This temperature increase was much faster than that in the in vitro experiments. One possible reason is that the concentration of SPD increases relative to the original solution with the permeation of water in the tumor. Meanwhile, the thermal conductivity of the tumor is smaller than that of the aqueous solution, leading to more significant thermal aggregation. Video S3 (Supporting Information) shows temperature mapping of intratumoral SPD in the PTT process by using the estimation of the PA signal. These preliminary experimental results prove that the SPD-mediated PA signal has good performance in tumor enrichment and temperature response with a high signal-to-noise ratio.

**Fig. 8:**
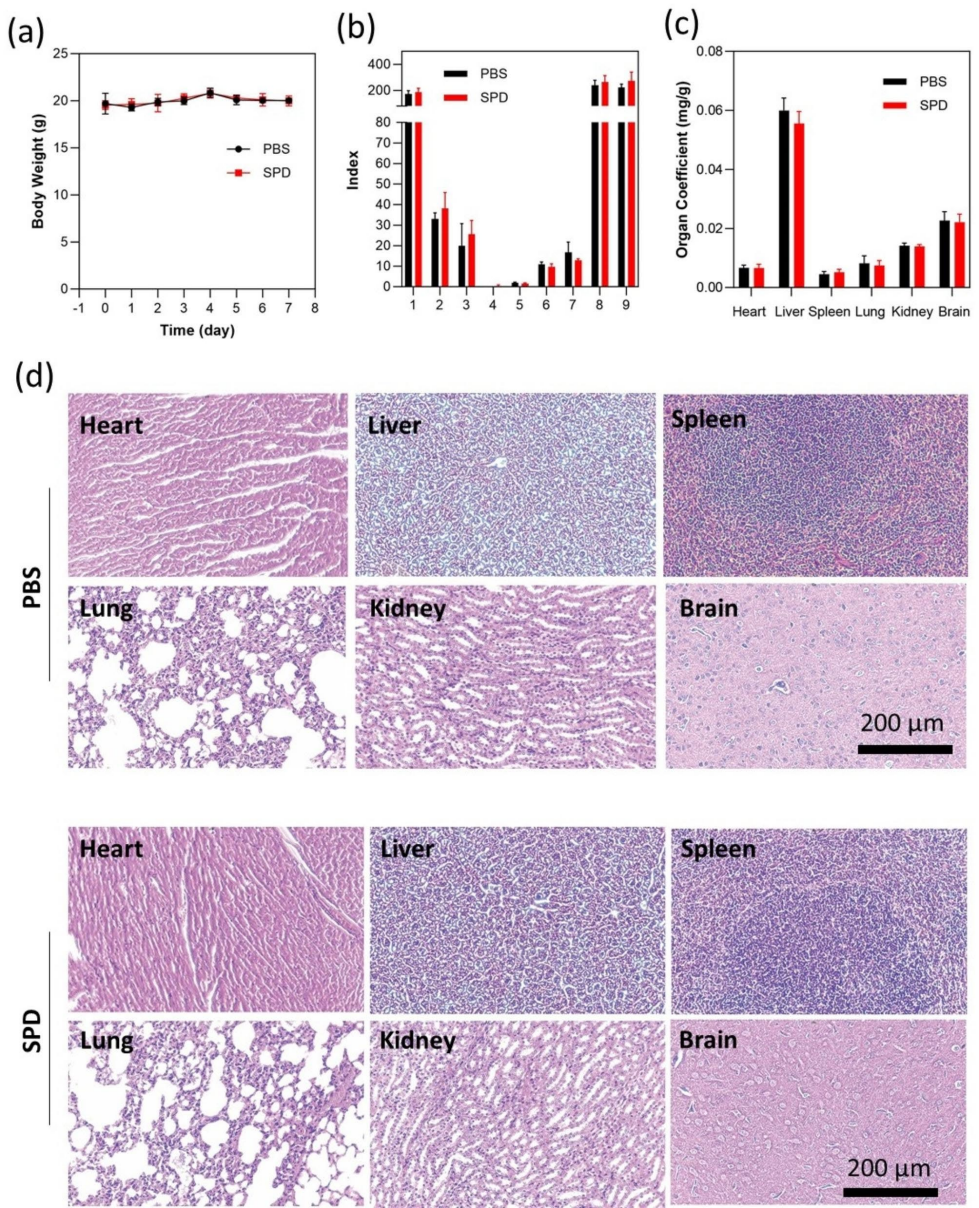
In vivo biocompatibility evaluation. (**a**) Body weight of BALB/C mice injected with PBS or SPD. (**b**) Blood biochemistry test of BALB/C mice injected with PBS or SPD after 7 days. (1: AST (U/L); 2: ALB II(g/L); 3: ALT (U/L); 4: GLU (mmol/L); 5: TG (mmol/L); 6: UREA (mmol/L); 7: CR (u mol/L); 8: ALP (U/L); 9: CK (U/L)) (**c**) Organ coefficients of major organs of BALB/C mice injected with PBS or SPD. (**d**) Histological analysis of major organs of BALB/C mice injected with PBS or SPD based on the HE staining

Finally, the in vivo biocompatibility of SPD was analyzed. As shown in Fig. 8a, the body weight of mice injected with either PBS or SPD remains steadily during the observation session and does not give a clear divide, indicating the negligible acute toxicity of SPD. The low hemolysis rate across a large concentration range of SPD demonstrates the bio-inertness of SPD to red blood cells (Fig. S9). On day 7, all mice were sacrificed, and the blood and major organs were harvested for further analysis. As presented in Fig. S10, all indexes of blood routine test related to red blood cells, white blood cells, and platelets remains nearly identical between the SPD group and the PBS group, implying that SPD would not induce infection, anemia, bleeding, and other symptoms in healthy mice. In addition, a similar trend has been observed in the blood biochemistry test results, indicating no liver and kidney dysfunctions (Fig. 8b). The organ coefficient values in Fig. 8c imply that SPD has a negligible effect on the state of major organs. Furthermore, the histological analysis of organ sections does not reveal inflammation or abnormalities in both the SPD group and the PBS group (Fig. 8d). Moreover, within the safety evaluation time, all mice behaved normally without any pathological differences in body shape, eating, and defecating. The above-mentioned results clearly confirm the safe use of SPD in biomedical applications.

## Discussion

Noninvasive thermometry is important for achieving successful thermal therapy with low side effects. Compared with the others, PA thermometry has the advantages of real-time, high sensitivity and spatial resolution [31], and good compatibility. In particular, with the development of nanosized PTAs, PA thermometry integrated with PTT and PAI will lead to more efficiency and flexibility for cancer treatments [32]. There are many reports about the integration of PTT and PAI. Unfortunately, very few works on the integration of PA thermometry and PTT have been reported, and most of reports use ITC for temperature detection, which can only reflect the temperature of the surface rather than the heat source deep in situ. Although the correlation between PA signals and the temperature of the AuNRs in the PTT process was discussed by Wang et al., the limited measuring depth by using an 808 nm laser wavelength cannot reflect the advantages of PA thermometry over ITC [33]. On the other hand, the measurement accuracy and stability are relatively poor due to the instability of AuNRs under the nanosecond pulse laser. Chen et al. proved that silica-coated AuNRs could promote the sensitivity of the PA temperature mapping [34]. However, our results showed that the PA amplitude of silica-coated AuNRs decreased significantly within 23 laser pulses, and the final amplitude was only 20% of the original amplitude. Zheng et al. used the dye aggregates with the conformational lability at different temperatures to confer temperature threshold sensing [35]. However, such a method has difficulty monitoring the continuous temperature changes, and the imaging depths will be limited by the short absorption peak of the dye aggregates. More recently, Sun et al. constructed a polyethylene glycol-coated tungsten-doped vanadium dioxide PA nanothermometer, which can linearly and reversibly respond to the thermal field near the human-body-temperature range (35–45 °C) [18]. However, it still has shortage in narrow measurement range and potential risk of heavy metal.

Here, we have fabricated SPD as a new nanosized PTA that exhibits outstanding features for both PTT and PA thermometry applications, such as high photothermal conversion efficiency, enhanced tissue penetration, and improved biocompatibility and targeting potential. According to the Jablonski diagram, the energy relaxation process generally includes fluorescence emission, heat generation, and triplet exciton generation. Considering the planar and rigid structure of NDTA and TDPP, strong intermolecular interactions (π-π stacking) occur during the nanoprecipitation process, thus leading to the diminished energy channel for radiative decay based on the aggregation-caused fluorescence quenching effect. In addition, attributed to the strong electron-withdrawing ability of TDPP and the long conjugation length along with the polymer chain, NT polymer is expected to have strong intramolecular charge transfer. Moreover, six carbonyl groups and two cyano groups on each unit of NT polymer afford vigorous stretching vibrations, which are decidedly conducive to the intramolecular motion in the aggregated state and highly promising to support heat generation. Furthermore, the time-dependent DFT calculation reveals that NT polymer has large ΔEst (0.454), which is not favorable for intersystem crossing (ISC) and triplet exciton generation. These calculation results are well in line with the expectation of polymer design. Thus, SPD with efficient photothermal generation, diminished fluorescence emission, and low ISC rate is a promising photothermal agent. Indeed, the SPD-mediated PA thermometry can also play an important role in controlled drug release, neurothermal regulation, and the mechanism of temperature-related biological function.

As temperature monitoring, photoacoustic detection has emerged for the visualization of PTAs and PTT. However, the measurement accuracy and tissue penetration depth of PA thermometry are still the limitations in deep tissue. In the subsequent work, more highly sensitively ultrasound transducer can be used to improve temperature measurement accuracy. For imaging depth, yet the strong optical attenuation of biological tissue has traditionally prevent PA imaging depth at several centimeters. Some methods can been developed to extend the imaging depth. For example, besides the strategy to develop the PTAs in the NIR-II window, novel imaging system such as internal illumination to deliver light into the inside organ, and imaging processing method such as optical fluence compensation algorithms, can be used to obtain weaker signals at larger depths. As a proof of concept, the therapeutic outcomes of photothermal cancer treatment were not included in this study. For the accuracy of temperature control, we still need to establish a feedback system from the PA thermometry to the photothermal treatment.

## Conclusions

Our results demonstrate that the SPD has dual photostability under pulsed laser and continuous-wave laser irradiation in NIR II. Meanwhile, a strong correlation between the PA signal and the actual temperature of SPD can be observed with deep-tissue penetration. Compared with ITC, SPD-mediated PA thermometry has high precision, fast response and good reliability for temperature feedback. Preliminary experimental results in vivo show that the SPD-mediated photoacoustic signal has a high signal-to-noise ratio, as well as good performance in temperature response and tumor enrichment. Such a study not only offers a new nanomaterial for the visualization of PTT at the treatment site and temperature feedback, but will also promote the theranostic platform for clinical applications.

### Electronic supplementary material

Below is the link to the electronic supplementary material.

Supplementary Material 1: **Figure S1-S3.** 1H NMR spectrum, GPC measurement and the absorption spectrum of NT polymer. **Figure S4.** The zeta potential of SPD. **Figure S5.** The heat conversion efficiency analysis of SPD. **Figure S6.** The TEM image and absorption spectrum of silica-coated AuNRs. **Figure S7.** Statistics of temperature distribution detected by ITC. **Figure S8.** The simulation of heat diffusion by using k-Wave. **Figure S9.** Hemolysis rate of SPD across diverse concentrations. **Figure S10.** Blood routine test

Supplementary Material 2: The video of temperature distribution of the SPD solution detected by ITC in the cyclic PTT process

Supplementary Material 3: The video of temperature distribution of a heating plate that is partially covered with a 9 mm thickness phantom by using ITC

Supplementary Material 4: The video of temperature mapping of intratumoral SPD in PTT process, which was constructed by the estimation of PA signal

## Data Availability

The collected and analyzed datasets during this study are available from the corresponding author on reasonable request.

## References

[1] Arami H, Kananian S, Khalifehzadeh L (2022). Remotely controlled near-infrared-triggered photothermal treatment of brain tumours in freely behaving mice using gold nanostars. Nat Nanotechnol.

[2] Hu Y, Chi C, Wang S (2017). A comparative study of clinical intervention and interventional photothermal therapy for Pancreatic cancer. Adv Mater.

[3] Chu KF, Dupuy DE (2014). Thermal ablation of tumours: Biological mechanisms and advances in therapy. Nat Rev Cancer.

[4] Chang M, Hou Z, Wang M (2021). Recent advances in hyperthermia therapy-based synergistic immunotherapy. Adv Mater.

[5] Yang Z, Gao D, Zhao J (2023). Thermal immuno-nanomedicine in cancer. Nat Rev Clin Oncol.

[6] Miller IC, Zamat A, Sun LK (2021). Enhanced intratumoural activity of CAR T cells engineered to produce immunomodulators under photothermal control. Nat Biomed Eng.

[7] Huang L, Li Y, Du Y (2019). Mild photothermal therapy potentiates anti-PD-L1 treatment for immunologically cold tumors via an all-in-one and all-in-control strategy. Nat Commun.

[8] Chouhan B, Dasgupta PK (2019). Direct photothermal measurement of optical absorption in a flow system. Anal Chem.

[9] Xu Z, Zhang Y, Zhou W (2021). NIR-II-activated biocompatible hollow nanocarbons for cancer photothermal therapy. J Nanobiotechnol.

[10] Kocincova AS, Borisov SM, Krause C, Wolfbeis OS (2007). Fiber-optic microsensors for simultaneous sensing of oxygen and pH, and of oxygen and temperature. Anal Chem.

[11] Lewis MA, Staruch RM, Chopra R (2015). Thermometry and ablation monitoring with ultrasound. Int J Hyperthermia.

[12] Gupta D, Choi D, Lu N (2023). Magnetic resonance thermometry targeting for magnetic resonance-guided histotripsy treatments. Ultrasound Med Biol.

[13] Larina IV, Larin KV, Esenaliev RO (2005). Real-time optoacoustic monitoring of temperature in tissues. J Phys D Appl Phys.

[14] Yan Y, John S, Shaik T (2021). Photoacoustic-guided endovenous laser ablation: characterization and in vivo canine study. Photoacoustics.

[15] Shah J, Park S, Aglyamov S (2008). Photoacoustic imaging and temperature measurement for photothermal cancer therapy. J Biomed Opt.

[16] Basij M, John S, Bustamante D (2023). Integrated ultrasound and photoacoustic-guided laser ablation theranostic endoscopic system. IEEE Trans Biomed Eng.

[17] Ma Y, Liu Y, Qin Z (2023). Mild-temperature photothermal treatment method and system based on photoacoustic temperature measurement and control. Biomed Signal Proces.

[18] Sun T, Zhang Z, Cui D (2023). Quantitative 3D temperature rendering of deep tumors by a NIR-II reversibly responsive W-VO2@PEG photoacoustic nanothermometer to promote precise cancer photothermal therapy. ACS Nano.

[19] Zhu H, Li B, Yu Chan C (2023). Advances in single-component inorganic nanostructures for photoacoustic imaging guided photothermal therapy. Adv Drug Deliv Rev.

[20] Huang X, Zhang W, Guan G (2017). Design and functionalization of the NIR-responsive photothermal semiconductor nanomaterials for cancer theranostics. Acc Chem Res.

[21] Chen YS, Zhao Y, Yoon SJ (2019). Miniature gold nanorods for photoacoustic molecular imaging in the second near-infrared optical window. Nat Nanotechnol.

[22] Cavigli L, Khlebtsov BN, Centi S (2021). Photostability of contrast agents for photoacoustics: the case of gold nanorods. Nanomaterials.

[23] Singh S, Giammanco G, Hu CH (2023). Size-tunable ICG-based contrast agent platform for targeted near-infrared photoacoustic imaging. Photoacoustics.

[24] Jiang Y, Tan Z, Zhao T (2023). Indocyanine green derived carbon dots with significantly enhanced properties for efficient photothermal therapy. Nanoscale.

[25] Kruger RA, Reinecke DR, Kruger GA (1999). Thermoacoustic computed tomography-technical considerations. Med Phy.

[26] Kim J, Choi W, Park EY (2019). Real-time photoacoustic thermometry combined with clinical ultrasound imaging and high-intensity focused ultrasound. IEEE Trans Biomed Eng.

[27] Pramanik M, Wang LV (2009). Thermoacoustic and photoacoustic sensing of temperature. J Biomed Opt.

[28] Lin X, Shen Y, Wang L (2021). Multi-scale photoacoustic assessment of wound healing using chitosan-graphene oxide hemostatic sponge. Nanomaterials.

[29] Chen P, Ma Y, Zheng Z (2019). Facile syntheses of conjugated polymers for photothermal tumour therapy. Nat Commun.

[30] Treeby BE, Cox BT (2010). k-Wave: MATLAB toolbox for the simulation and reconstruction of photoacoustic wave fields. J Biomed opt.

[31] Gao L, Wang L, Li C (2013). Single-cell photoacoustic thermometry. J Biomed Opt.

[32] Moore C, Jokerst JV (2019). Strategies for image-guided therapy, Surgery, and drug delivery using photoacoustic imaging. Theranostics.

[33] Wang SH, Wei CW, Jee SH (2011). Quantitative thermal imaging for plasmonic photothermal therapy. J Med Biol Eng.

[34] Chen YS, Frey W, Walker C et al. (2013) Sensitivity enhanced nanothermal sensors for photoacoustic temperature mapping. J Biophotonics 2013; 6(6–7): 534–542. 10.1002/jbio.201200219 23450812

[35] Ng KK, Shakiba M, Huynh E (2014). Stimuli-responsive photoacoustic nanoswitch for in vivo sensing applications. ACS Nano.

