# Supplementary material for: Visualization of photothermal therapy by semiconducting polymer dots mediated photoacoustic detection in NIR II
Source: J Nanobiotechnology. 2023 Dec 7;21:468. doi: 10.1186/s12951-023-02243-0 (PMC10701955; doi:10.1186/s12951-023-02243-0)
Supplement: Supplementary file 4 — Supplementary Material 4: The video of temperature mapping of intratumoral SPD in PTT process, which was constructed by the estimation of PA signal [file 12951_2023_2243_MOESM4_ESM.docx]

**Visualization of photothermal therapy by semiconducting polymer dots mediated photoacoustic detection in NIR II**

Xiangwei Lin^1#^, Zhourui Xu^1#^, Jiangao Li^2^, Hongji Shi^1^, Zhenyu Fu^1^, Yuqing Chen^1^, Wenguang Zhang^1^, Yibin Zhang^1^, Haoming Lin^1^, Gaixia Xu^1^, Xin Chen^1^, Siping Chen^1^, Mian Chen^1*^

1 National-Regional Key Technology Engineering Laboratory for Medical Ultrasound, Guangdong Key Laboratory for Biomedical Measurements and Ultrasound Imaging, School of Biomedical Engineering, Shenzhen University Medical School, Shenzhen University, Shenzhen, 518055 China

2 Center for AIE Research, Shenzhen Key Laboratory of Polymer Science and Technology, College of Material Science and Engineering, Shenzhen University, Shenzhen, 518060, China

**Supporting Figures**


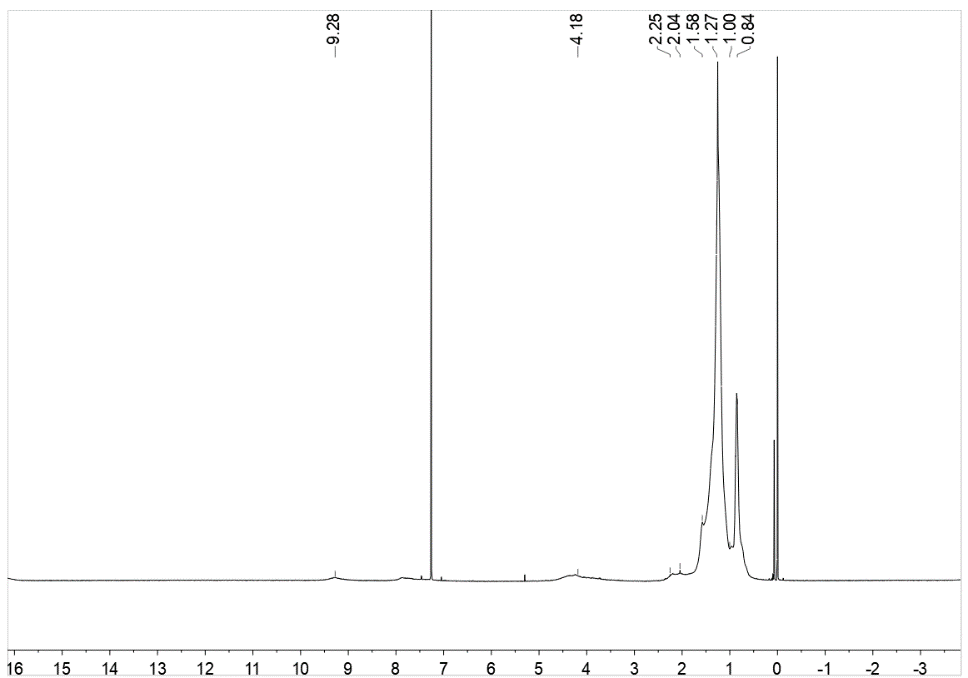


**Figure S1**. ^1^H NMR spectrum of NT polymer.


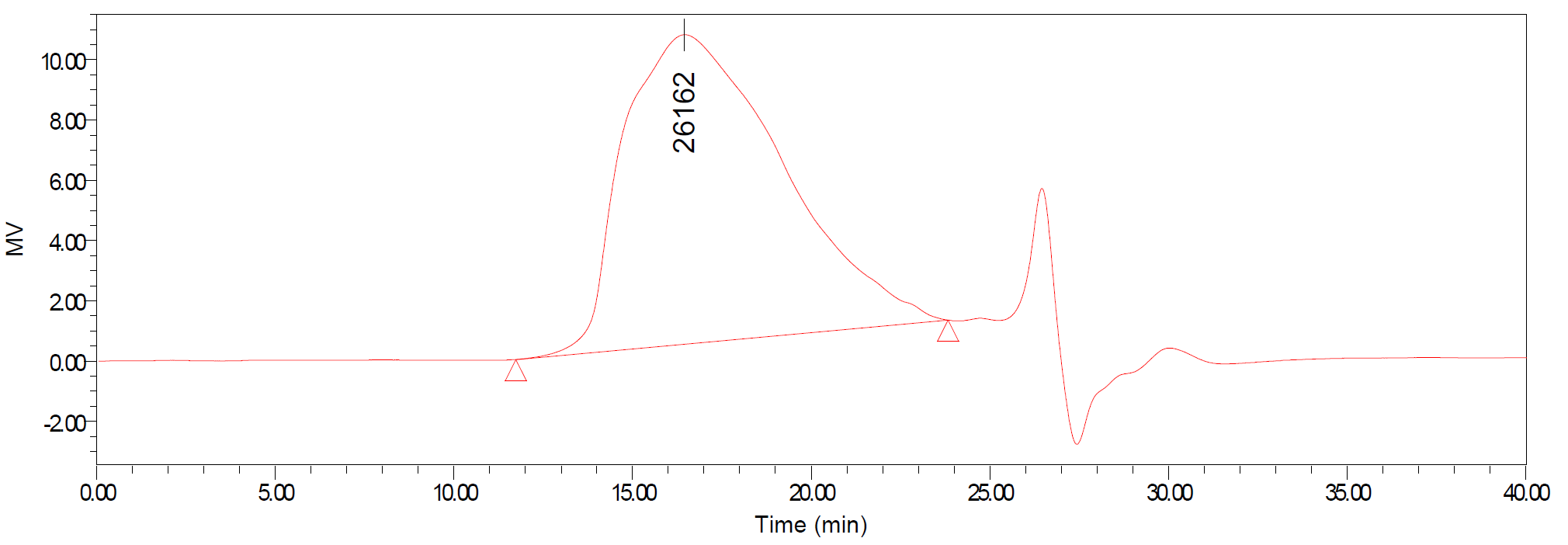


**Figure S2**. GPC measurement of NT polymer, using THF as eluent and polystyrene as a standard. Mn: 13484 g/mol, PD: 2.26.

**
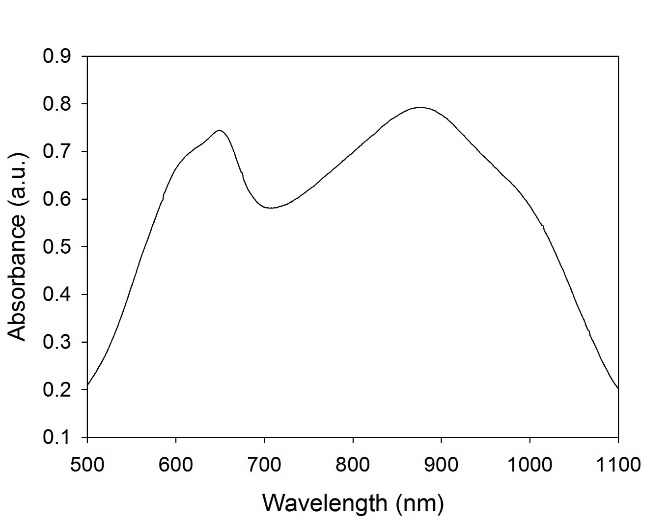
**

**Figure S3.** The absorption spectrum of NT polymer in tetrahydrofuran solution.


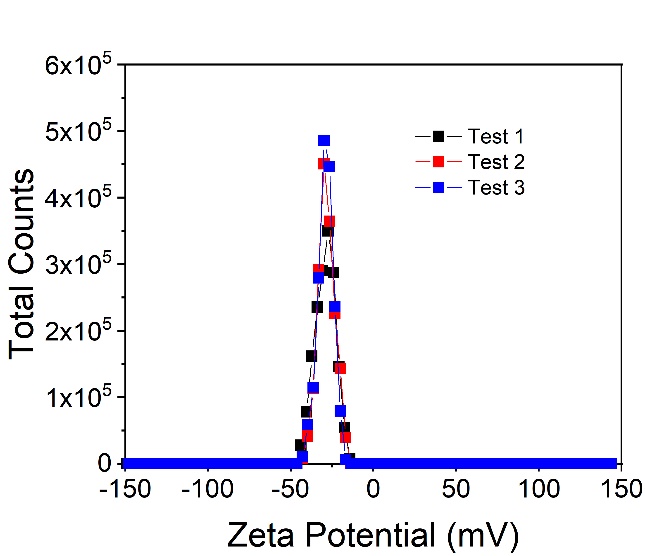


**Figure S4.** The zeta potential of SPD in aqueous solution.


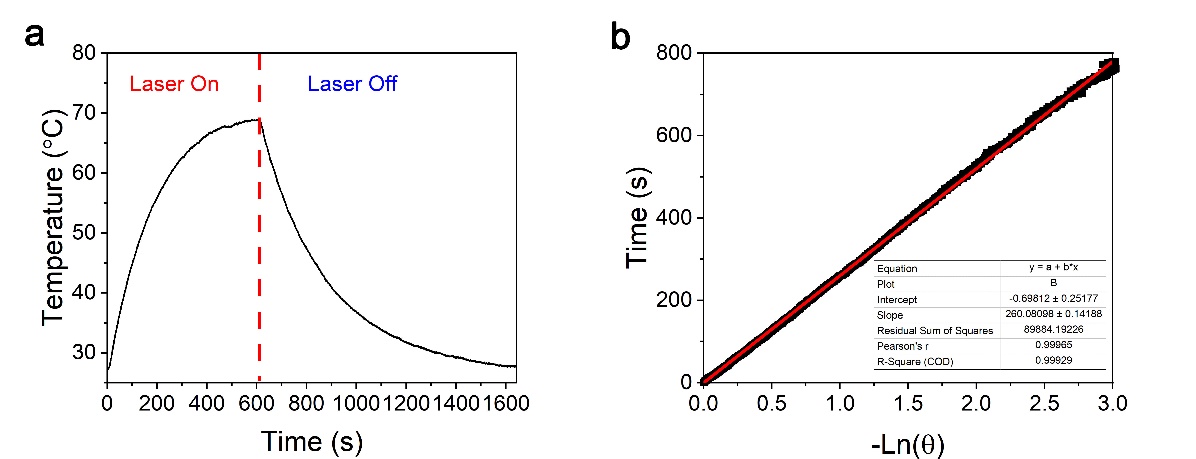


**Figure S5.** The heat conversion efficiency analysis of SPD. (a) Lasering and natural cooling curve of SPD. (b) Time constant for heat transfer from the system was determined to be τ_s_ =260.1s.


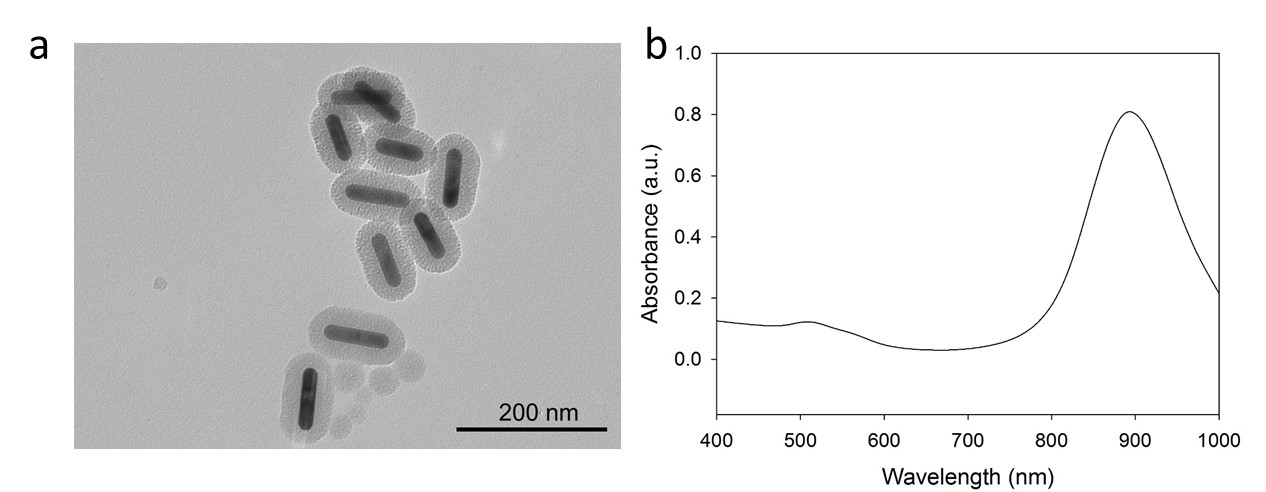


**Figure S6.** (a) TEM image and (b) absorption spectrum of silica-coated AuNRs.


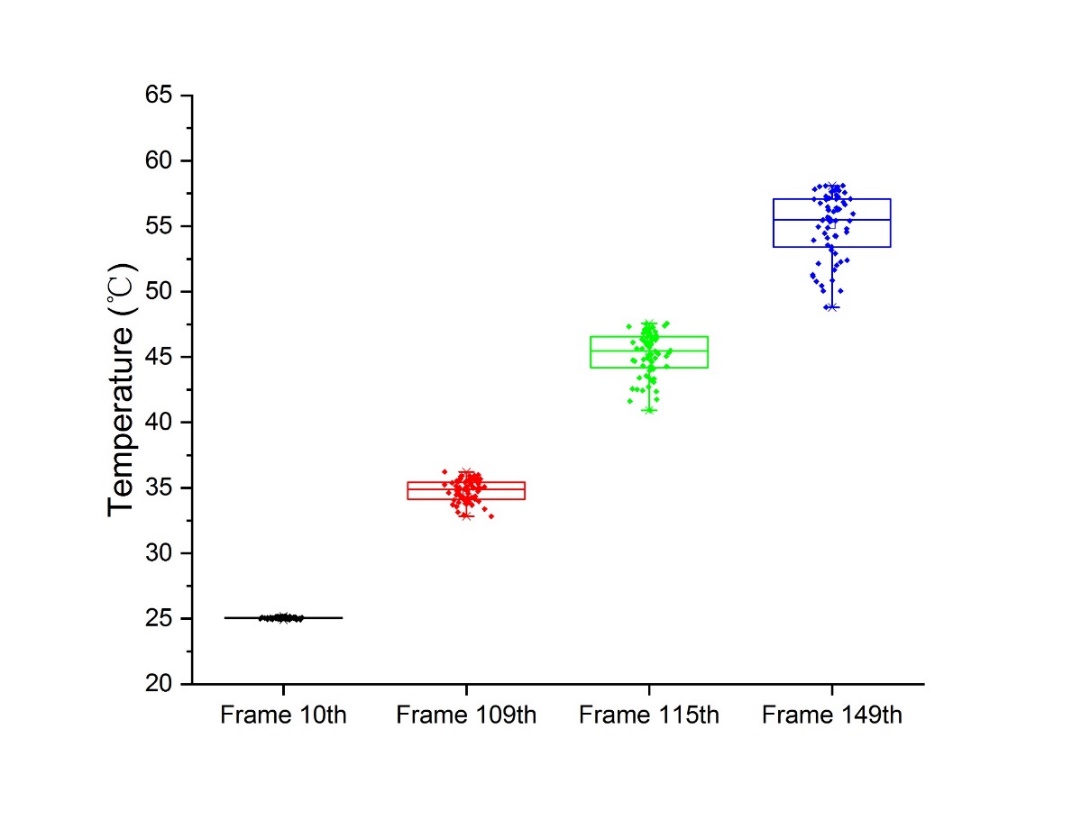


**Figure S7.** Statistics of temperature distribution detected by ITC in the ROI area with 13 × 5 pixels.


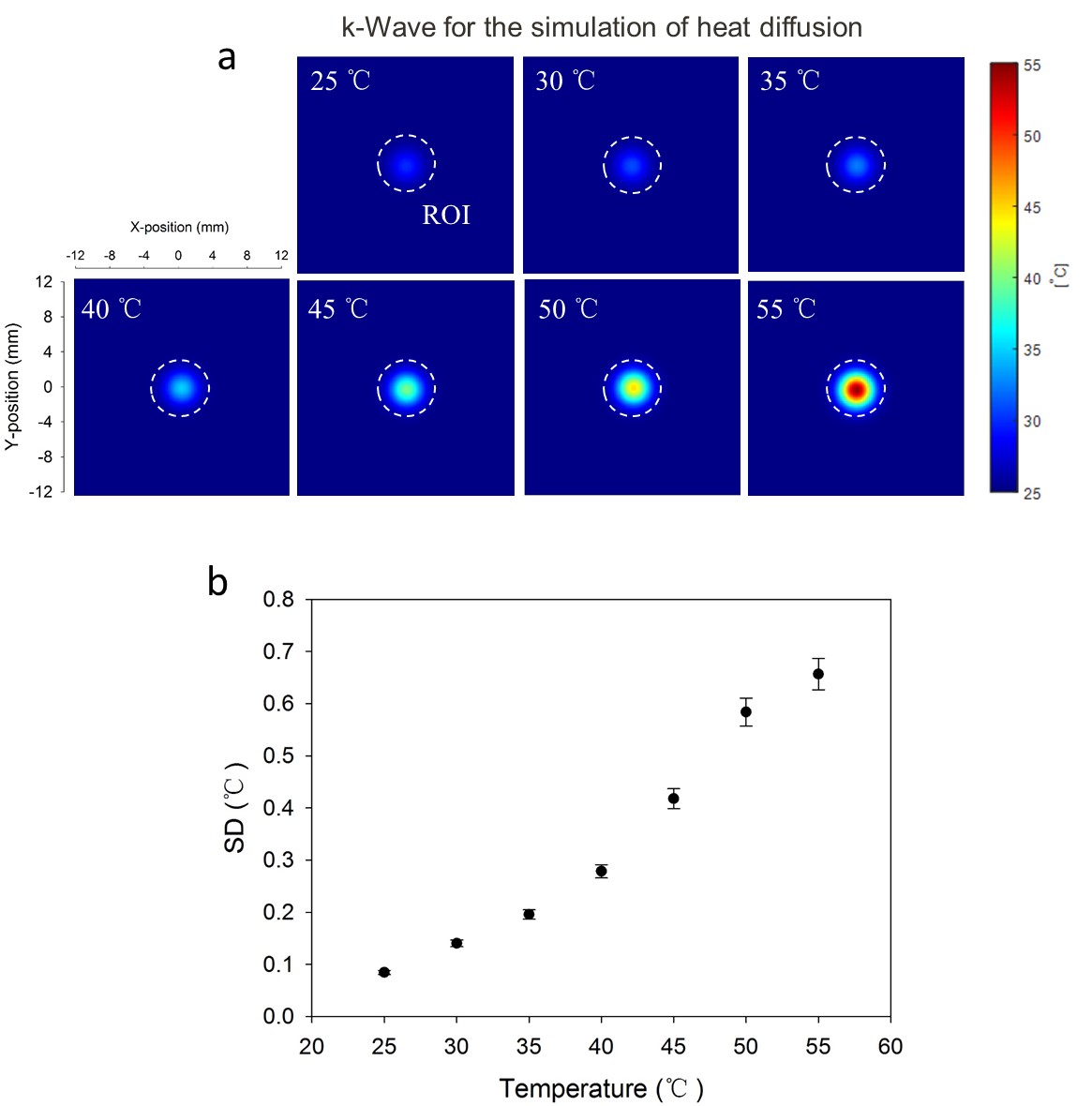


**Figure S8.** (a) The simulation of heat diffusion by using k-Wave. (b) The standard deviation (SD) of temperature distribution in the ROI area from k-Wave simulation.


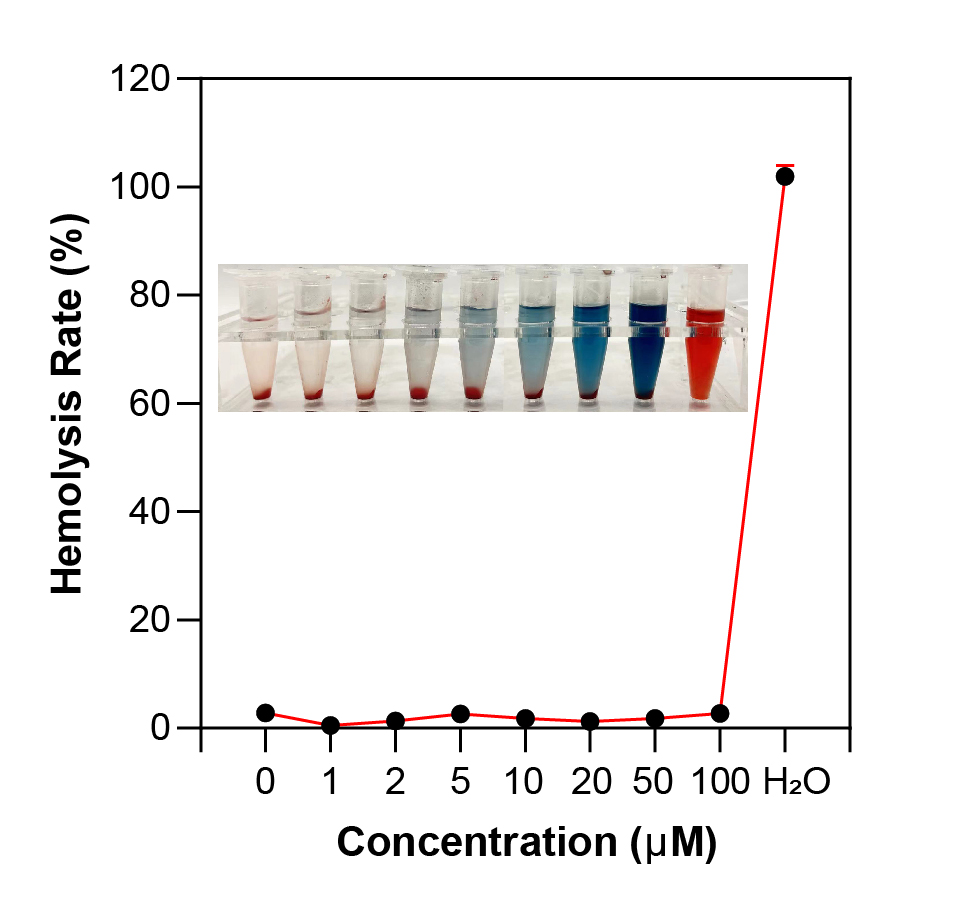


**Figure** **S9**. Hemolysis rate of SPD across diverse concentrations.


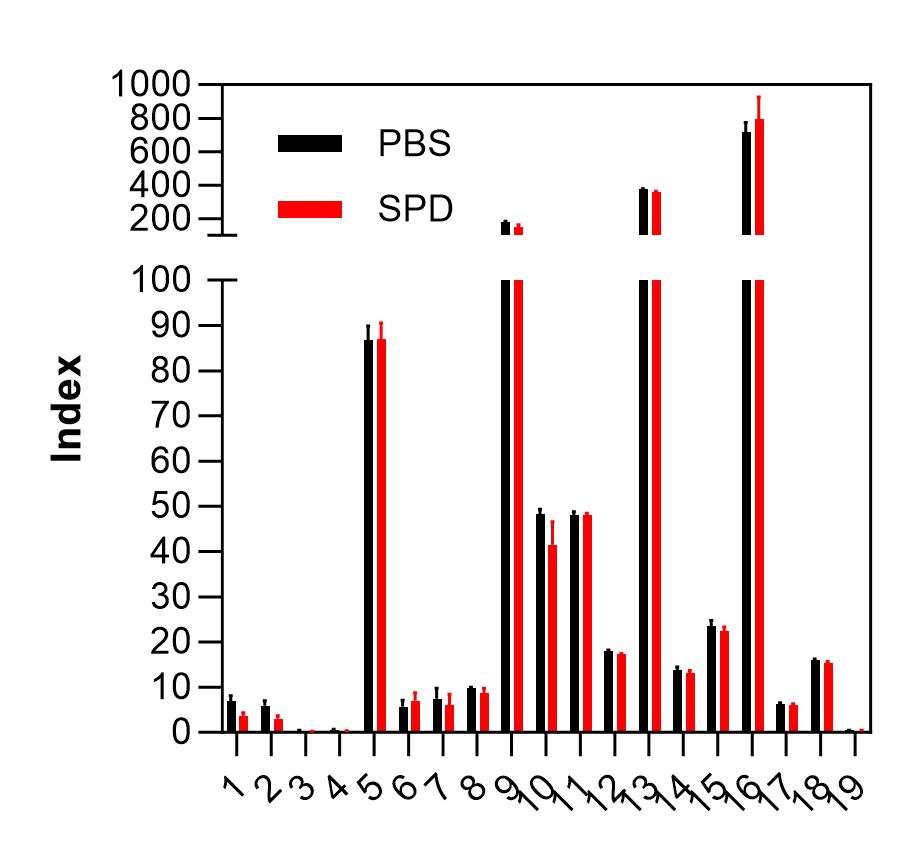


**Figure S10**. Blood routine test of BALB/C mice injected with PBS or SPD after 7 days. (1:WBC (10^^9^/L); 2: Lymph# (10^^9^/L); 3: Mid# (10^^9^/L); 4” Gran# (10^^9^/L); 5: Lymph% (%); 6: Mid% (%); 7: Gran% (%); 8: RBC (10^^12^/L); 9: HGB (g/L); 10: HCT (%); 11: MCV (fL); 12: MCH (pg); 13: MCHC (g/L); 14: RDW-CV (%); 15: RDW-SD (fL); 16: PLT (10^^9^/L); 17: MPV (fL); 18: PDW; 19: PCT (%))

**Supporting Videos**

SI Video 1.

The temperature imaging of the SPD solution detected by ITC in the cyclic PTT process. The video was captured at 3 fps and was played at 30 fps.

SI Video 2.

The temperature imaging of a heating plate that is partially covered with a 9 mm thickness phantom by using ITC. The video was captured at 3 fps and was played at 3 fps.

SI Video 3.

The temperature mapping of intratumoral SPD in PTT process, which was constructed by the estimation of PA signal. EG: Experimental group, which was under irradiation by 1064 nm CW laser. CG: Control group, which was without irradiation by 1064 nm CW laser. The video was played at 0.5 fps.
